# 1,3-Diphenylethenylcarbazolyl-Based Monomer for Cross-Linked Hole Transporting Layers

**DOI:** 10.3390/molecules20059124

**Published:** 2015-05-19

**Authors:** Maryte Daskeviciene, Giedre Bubniene, Tadas Malinauskas, Vygintas Jankauskas, Valentas Gaidelis, Valdas Paulauskas, Vytautas Getautis

**Affiliations:** 1 Department of Organic Chemistry, Kaunas University of Technology, Radvilenu pl. 19, 50254 Kaunas, Lithuania; E-Mails: maryte.daskeviciene@ktu.lt (M.D.); giedre.bubniene@gmail.com (G.B.); tadas.malinauskas@ktu.lt (T.M.); 2 Department of Solid State Electronics, Vilnius University, Sauletekio 9, 10222 Vilnius, Lithuania; E-Mails: vygintas.jankauskas@ff.vu.lt (V.J.); valentas.gaidelis@ff.vu.lt (V.G.); 3 Institute of Environment and Ecology, Aleksandras Stulginskis University, Studentu 11, Kaunas Dist., 53361 Akademija, Lithuania; E-Mail: valdas.paulauskas@asu.lt

**Keywords:** cross-linking, carbazole, epoxide, molecular glass, hole drift mobility, ionization potential

## Abstract

A new cross-linkable monomer containing 1,3-diphenylethenylcarbazolyl-based hole-transporting moieties and four reactive epoxy groups, was prepared by a multistep synthesis route from 1,3-bis(2,2-diphenylethenyl)-9*H*-carbazol-2-ol and its application for the *in situ* formation of cross-linked hole transporting layers was investigated. A high concentration of flexible aliphatic epoxy chains ensures good solubility and makes this compound an attractive cross-linking agent. The synthesized compounds were characterized by various techniques, including differential scanning calorimetry, xerographic time of flight, and electron photoemission in air methods.

## 1. Introduction

Electronic and optoelectronic devices using organic materials as active elements, for example organic light-emitting diodes (OLED), organic photovoltaic devices (OPV), organic field-effect transistors (OFET), organic photoreceptors, organic photorefractive devices and so forth, have received a great deal of attention from the standpoint of potential technological applications as well as fundamental science [1,2,3,4,5]. All the devices described above involve charge transport as an essential operation process and hence, require proper charge-transporting materials. In order to ensure high efficiency multi-layered devices containing charge (hole and electron) injection and transporting layers are usually fabricated. Fabrication is usually carried out using one of two methods: high vacuum vapor deposition for small molecules or solution processing for both polymers and small molecules. Multilayer structures could be constructed through layer-by-layer vapor deposition, a very successful technique that is however limited to thermally stable low-molecular-weight materials and is relatively expensive and time-consuming. Fabrication of the devices from solution would be an attractive alternative. The process is non-trivial however, as one particular requirement for the multilayer coating is resistance of the previously coated layer to the solvent used to deposit a subsequent one [6]. Several strategies have been developed to overcome this technological problem and perhaps the most elegant one involves development of the cross-linkable materials. They can be solution processed by doctor blading or ink jet printing and then transformed into an insoluble film by light [7,8], heat [9,10,11] or chemical [12] treatment. Therefore, development of the high-performance, cross-linkable charge-transporting materials are a key issue for the fabrication of efficient devices.

Novel carbazole-based derivatives possessing diphenylethenyl fragments have been reported by us recently [13]. The commercial availability and relative cheapness of the starting materials, simple synthetic method and a number of sites available for easy functionalization, good charge drift mobility and solubility in common organic solvents makes these precursors attractive building blocks for the construction of more complex materials for optoelectronic applications [14]. Herein, we present the synthesis of a 1,3-diphenylethenylcarbazolyl-based monomer as well as its application in the fabrication of the cross-linked hole transporting layers. High concentration of flexible aliphatic epoxy chains ensures good solubility and makes this compound a very attractive cross-linking agent. Only a difunctional second component is necessary to ensure successful cross-linking, broadening the assortment of possible cross-linking agents considerably.

## 2. Results and Discussion

**Scheme 1:**
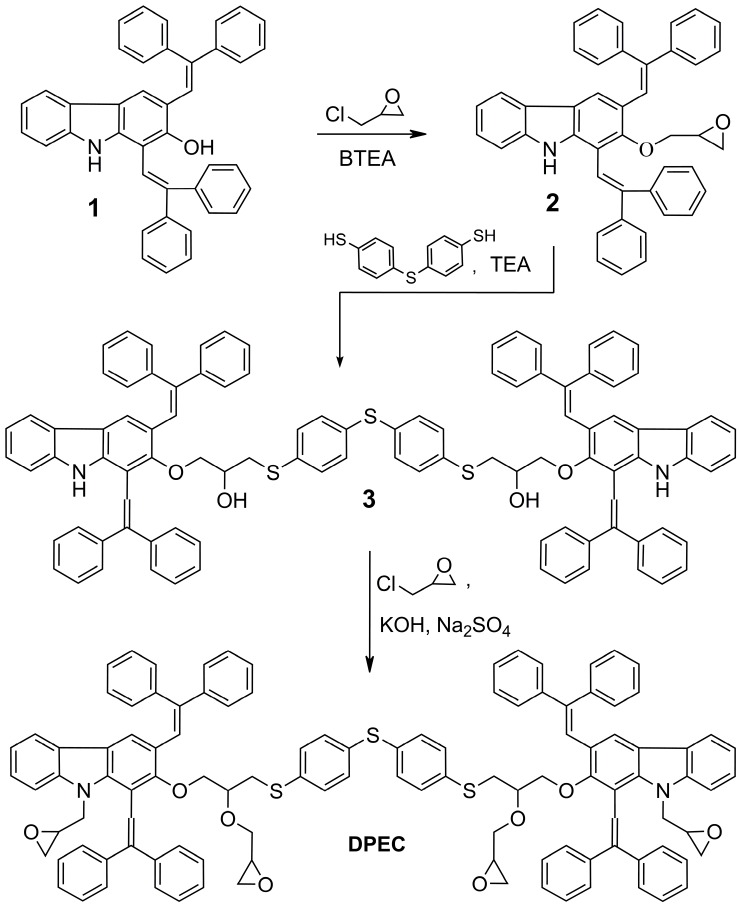
Synthesis of 1,3-diphenylethenylcarbazole-based monomer **DPEC**.

**Figure 1:**
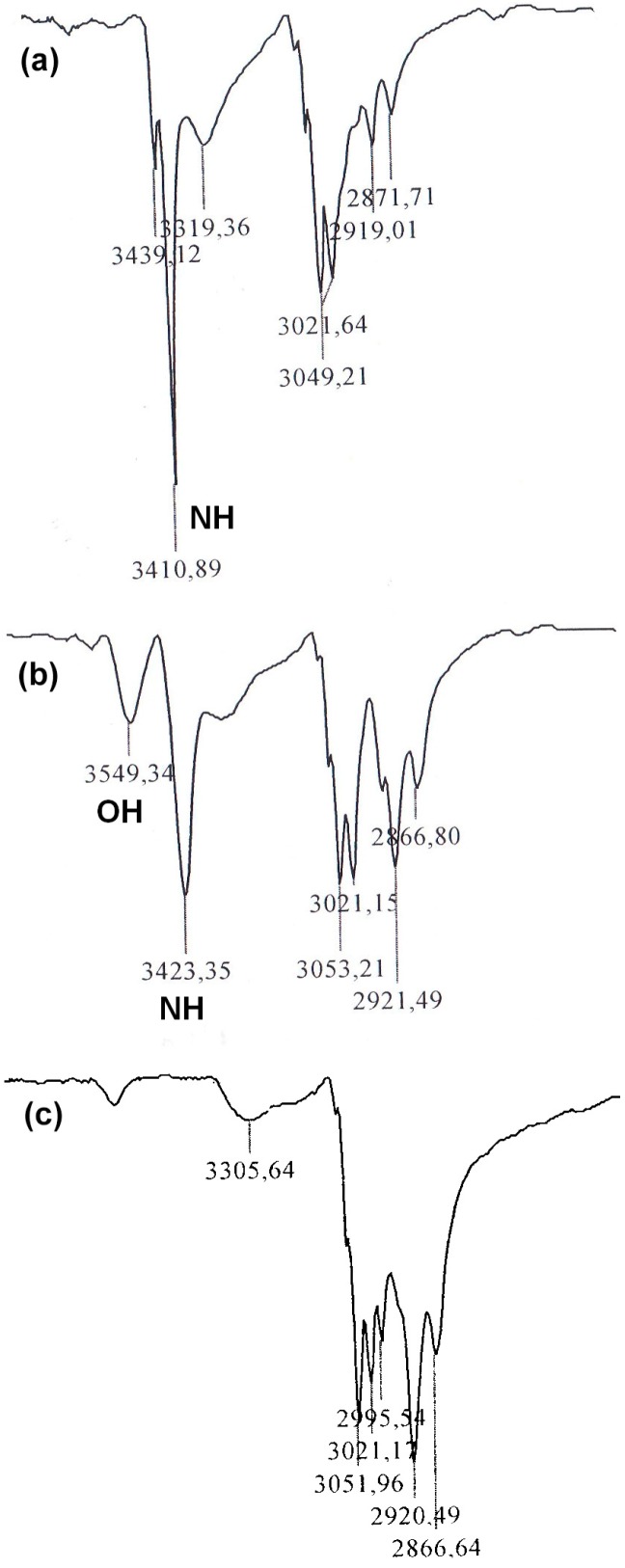
Fragments of the IR spectra of epoxide **2** (**a**), dimer **3** (**b**) and **DPEC** (**c**).

The three-step synthesis route to the four epoxy group-containing 1,3-diphenylethenylcarbazole-based monomer **DPEC** is shown in Scheme 1. 1,3-Bis(2,2-diphenylethenyl)-9*H*-carbazol-2-ol (**1**) was used as a starting compound in the synthesis of dimers possessing both hole transporting capabilities as well as four reactive epoxy groups for cross-linking purposes. It was determined that in the presence of benzyltriethylammonium chloride (BTEA) as a catalyst, only the aromatic hydroxyl group was alkylated and, consequently, 1,3-bis(2,2-diphenylethenyl)-2-(oxiran-2-methyl)-9*H*-carbazole (**2**) was isolated. The ^1^H-NMR spectrum of **2** has proven the presence of an oxiranyl ring by displaying the clearly defined parts of two ABX systems of the non-equivalent geminal protons of the oxiranyl C*H*_2_ and OC*H*_2_ moieties. The resonances of the former protons appear as two doublets of doublets (dd) at 2.60 ppm and 2.75 ppm with *J*_AB_ = 5.0, *J*_AX_ = 2.6 and *J*_BX_ = 4.1 Hz due to the coupling with oxiranyl C*H*, while the OC*H*_2_ protons give two dd in the 4.15–4.30 ppm region (*J*_AB_ = 11.1, *J*_AX_ = 5.7 and *J*_BX_ = 4.0 Hz), respectively. The preservation of the NH group has been confirmed by an absorption band at 3411 cm^−1^ in the IR spectrum (Figure 1a). Twin molecule **3** was obtained *via* nucleophilic ring opening reaction of epoxy compound 2 with 4,4ʹ-thiobisbenzenethiol (**TBBT**) in the presence of triethylamine (TEA), according to the synthetic procedures reported by us previously [15,16,17]. The presence of a secondary hydroxyl has been confirmed by a doublet (*J* = 4.9 Hz) at 2.61 ppm in the ^1^H-NMR spectrum as well as by additional absorption band at 3549 cm^−1^ in the IR spectrum (Figure 1b). Finally, alkylation of both OH and NH groups of the intermediate **3** with epichlorohydrin in the presence of KOH and anhydrous Na_2_SO_4_ provided the target monomer **DPEC** containing four epoxy groups. Absence of OH and NH group signals in the IR spectrum (Figure 1c) of **DPEC**, and broad and strong absorption bands in 1250–1000 cm^−1^ region, indicating the presence of numerous ether groups, are unambiguous proof that all OH and NH groups have been successfully alkylated.

The final product was isolated by column chromatography, dissolved in toluene and precipitated with *n*-hexane. **DPEC** obtained by such a procedure was amorphous and all attempts to crystallize it were unsuccessful. Such a high morphological stability of the amorphous **DPEC** apparently can be explained by the existence of several diastereomers, which could neither be separated nor characterized individually, and the presence of the numerous flexible aliphatic chains.

**Figure 2:**
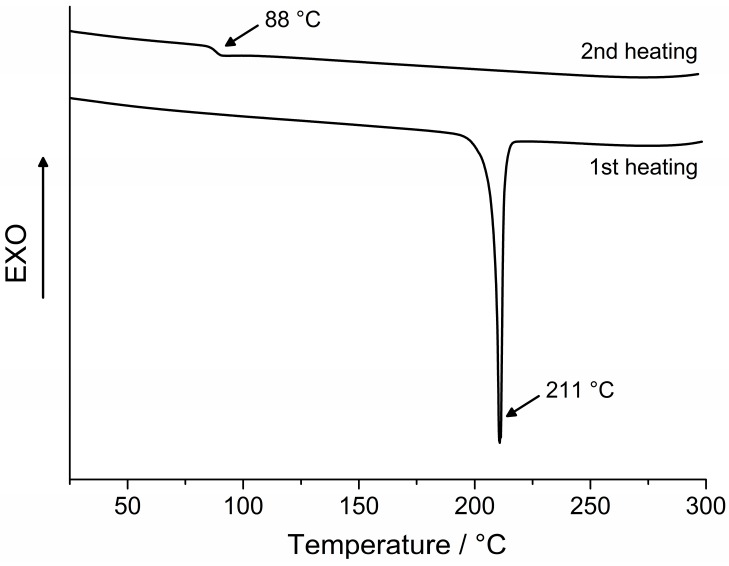
First and second heating DSC curves of **2** (heating rate 10 °C·min^−1^, N_2_ atmosphere).

**Figure 3:**
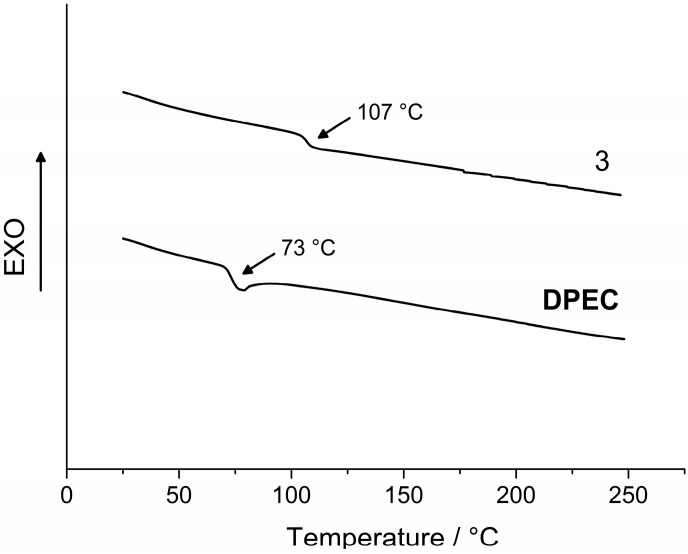
Second heating DSC curves of **3** and **DPEC** (heating rate 10 °C·min^−1^, N_2_ atmosphere).

The morphological properties of the synthesized compounds were studied by differential scanning calorimetry (DSC) and results are presented in Figure 2 and Figure 3 and Table 1. These investigations have revealed that intermediate **2** can exist both in crystalline and amorphous state, while dimers **3** and **DPEC** have been found only in an amorphous state in our experiments. The first heating DSC curve of **2** reveals melting at 211 °C, and no crystallization takes place during cooling or a second heating, and only a glass transition at 88 °C is registered during the second heating (Figure 2). The substance remains in glassy state after melting and subsequent cooling; therefore, monomer **2** can be considered to be a molecular glass. This is also a common feature for dimers 3 and **DPEC**, consisting of two chromophores linked by a central flexible bridge. Glass transition occurs at 107 °C for **3** and at 73 °C for **DPEC** (Figure 3), and no melting is observed for either of these structures, indicating that the original state of the sample was amorphous.

The comparison of obtained glass temperature (*T*_g_) values has revealed that both the molecular mass and nature of the functional groups have a significant influence on the glass transitions of the synthesized compounds. *T*_g_ was thus found to be higher for dimer **3** in comparison with the epoxy monomer **2** (107 °C and 88 °C, respectively). Replacement of hydrogen atoms by the epoxypropyl groups led to formation of glicidyl groups and changes in molecule’s conformation, which in turn decreased packing density of the **DPEC** molecules. These conformational changes and overall plastifying effect of the aliphatic chains determined a drop in *T_g_* from 107 °C for **3** to 73 °C for **DPEC**. 

**Table 1:** Thermal and photoelectrical properties of compounds **2**, **3**, **DPEC**, and cross-linked layer **NET1**.

Compound	*T_m_*, °C	*T_g_*, °C	*I_p_*, eV ^a^	μ_0_, cm^2^/V·s ^b^	μ, cm^2^/V·s ^c^
**2**	211	88	-	-	-
**3**	-	107	5.74	8 × 10^−8^^d^	2.5 × 10^−6^^d^
**DPEC**	-	73	5.77	3.6 × 10^−7^^d^	3.3 × 10^−6^^d^
**NET1**	-	-	5.65	6 × 10^−6^	3 × 10^−4^

^a^ Ionization potential (*I*_p_) was measured by the photoemission in air method from films; ^b^ Mobility value at zero field strength. ^c^ Mobility value at 1 × 10^6^ V·cm^−1^ field strength. ^d^ Measured in solid solution of hole transporting material in polycarbonate-Z (PC-Z), 1:1 w/w ratio.

**Figure 4:**
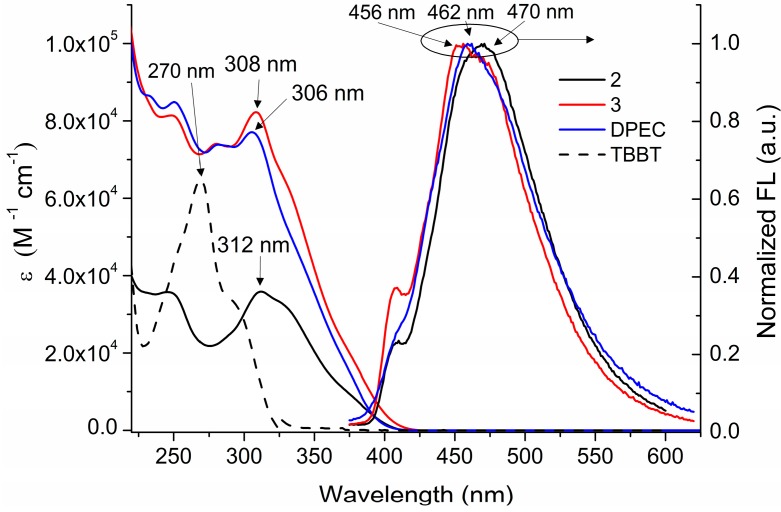
Absorption and emission spectra of dilute solutions of **TBBT**, **2**, **3**, and **DPEC**.

The optical properties of synthesized compounds were investigated by UV-Vis and fluorescence spectroscopy. All synthesized compounds exhibit a broad absorption in the 230–410 nm region (Figure 4). The light absorption spectra of the dimers **3** and **DPEC** consisting of two branches linked by the central flexible bridge are very similar to the one of monomeric structure **2**. Moreover, introduction of the **TBBT** linking fragment into the dimeric structures **3** and **DPEC** has been proven by the changes in the UV spectra around 275 nm region due to the presence of additional thiobisbenzene fragments. The comparison of UV spectra of compounds with one (compound **2**) and two (compounds **3** and **DPEC**) 1,3-diphenylethenylcarbazolyl moieties has shown that increasing number of these chromophores leads to a noticeable hyperchromic shift. Fluorescence of the synthesized HTMs is observed in the 400–620 nm region with a peak at ~460 nm. Emission spectra of the dimers **3** and **DPEC** are slightly blue shifted with respect to the mono-analogue 2, most likely due to slightly smaller size of the π-conjugated system in these molecules. The observed small hypsochromic shift is also observable in the absorption spectra and is most likely caused by a more crowded structure of the dimers **3** and **DPEC**.

**Figure 5:**
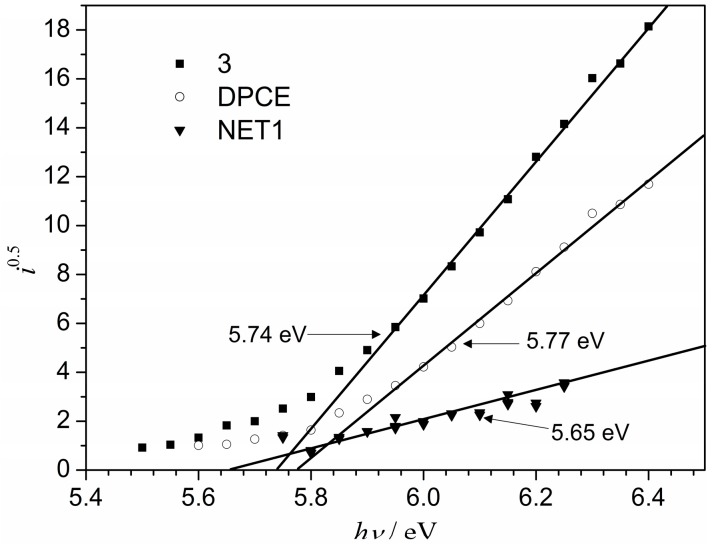
Photoemission spectra of the films of **3** and **DPEC**.

The electron photoemission spectra of the films of synthesized compounds **3** and **DPEC** are presented in Figure 5. Usually the photoemission experiments are carried out in vacuum and high vacuum is one of the main requirements for these measurements [18]. If the vacuum is not high enough, sample surface oxidation and gas adsorption will influence the measurement results. In our case, however, the organic substance investigated is stable enough to oxygen and the measurements may be carried out in air, furthermore, ionization potential (*I*_p_) values obtained in these measurements coincide with those achieved in vacuum [19]. The sample is prepared from solution in organic solvent in air, therefore O_2_ and other air gasses may not only be absorbed on the surface, but also saturate the volume of the sample. This organic layer is also used in practice, for example, in electrophotography, in air, so it is important to measure the characteristics of this material in air. The *I*_p_ values obtained by the electron photoemission in air method (5.74 eV for dimer 3 and 5.77 eV for tetraepoxy-based monomer **DPEC**) are close to the ones of the parent 1,3-diphenylethenylcarbazolyl-based compounds (5.62 eV) reported in [13]. A slight increase in the *I*_p_ could be attributed to the smaller π-conjugated systems in dimers **3** and **DPEC** due to increased structural crowding in these molecules.

**Figure 6:**
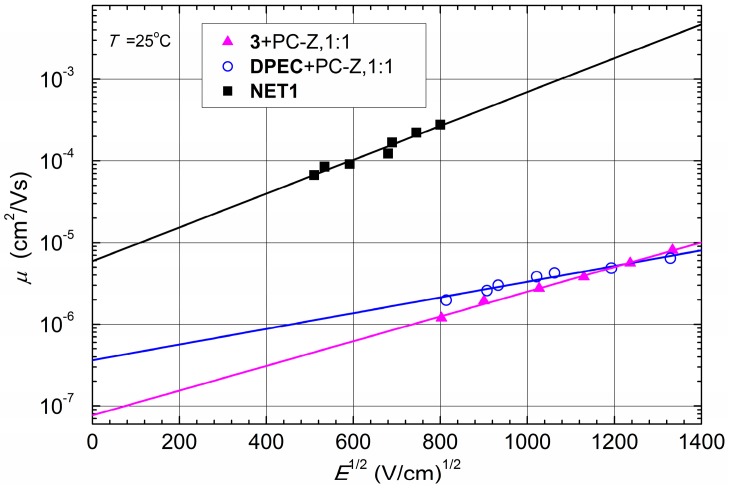
Electric field dependence of the hole drift mobility in charge transport layers of **3** and **DPEC** doped in PC-Z and in the cross-linked layer **NET1**.

**Figure 7:**
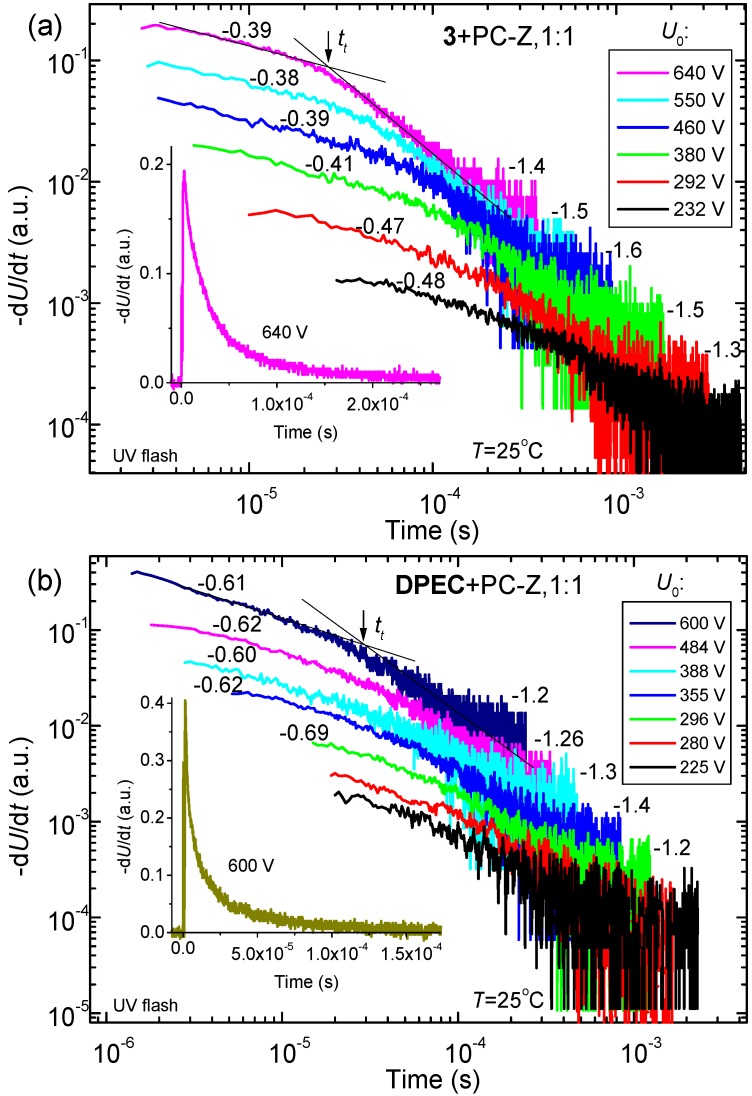
XTOF transients for the **3** (**a**) and **DPEC** (**b**). Insets show one transient curve in linear plot.

Compounds **3** and **DPEC** are soluble in common organic solvents such as chloroform, THF, dioxane, *etc.* This really good solubility is mainly due to the flexible aliphatic fragments. Clear, transparent and homogeneous films of **3** and **DPEC** in composition with PC-Z were obtained by the casting technique. The hole drift mobility for these molecular glasses was measured by xerographic time of flight technique (XTOF, Figure 6). XTOF measurements have revealed that small charge transport is not Gaussian, however transit time is seen on log-log plots in all investigated cases (Figure 7). The significantly dispersive character of the kinetics can be explained by large disorder in the investigated materials. Such dependencies of charge mobility on electrical field are characteristic for many organic photoconductors and are predicted by the Bässler-Borsenberger model [20,21]. As seen from the results of drift mobility presented in Figure 6 there is a small difference between the hole drift mobility values of compounds **3** and **DPEC** at strong electric fields, while at weak electric fields the hole drift mobility in about one order of magnitude higher. However, since the signal for **DPEC** is more dispersive than the one for compound **3** (Figure 7), the last conclusion should be interpreted with caution.

**DPEC**, containing four reactive epoxy groups, was utilized in the investigations aimed to develop cross-linked hole transporting layers with high solvent resistance and charge mobility high enough for many practical applications.

**Scheme 2:**
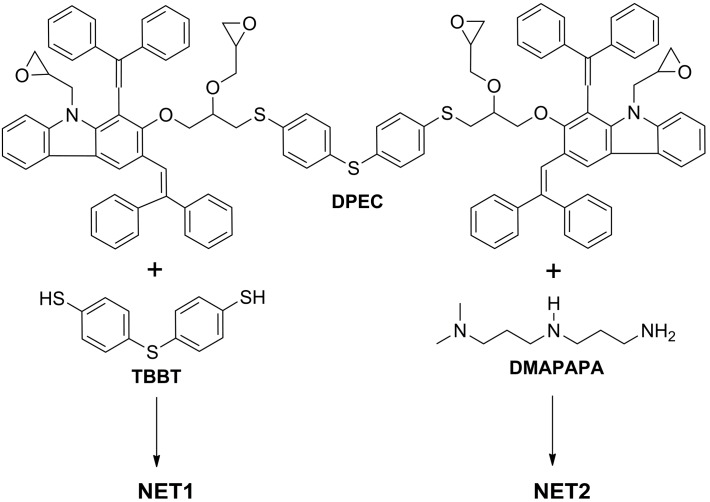
Composition of **NET1** and **NET2** cross-linking experiments.

It is well known [22], that epoxides react easily with nucleophilic reagents, particularly containing mercapto groups. Thus, in order to obtain a cross-linked film **NET1**, the reaction between tetrafunctional **DPEC** and a difunctional nucleophile **TBBT** was used (Scheme 2). Samples were coated on ITO and heated at 120 °C for 3 h. Solubility of the obtained **NET1** films was tested by measuring the thickness after treatment with THF. The control sample was stored under room conditions for the same duration. The results revealed that the control sample was soluble in THF, indicating that the layer was not cross-linked. On the other hand, the thermally treated layer was partially cross-linked. In order to accelerate the cross-linking process, the catalyst TEA was added to the composition prepared for the cross-linking experiments. Unfortunately, an unclear and opaque film was obtained before and after annealing. This negative result has indicated that all used components in this experiment are not compatible and this composition cannot cross-link. 

In order to obtain a cross-linked film **NET2**, the cross-linking agent **TBBT** was replaced by *N*,*N*-dimethyldipropylenetriamine (DMAPAPA), as aliphatic amines are very reactive and are usually used under ambient conditions (Scheme 2). A 3:4 molar ratio solution of **DPEC** with DMAPAPA was prepared in THF. Opaque layers were obtained before and after annealing at 120 °C, indicating that all used components are not compatible in this experiment. Therefore, an intermediate step was introduced in order to get clear layers. Initially, a soluble oligomer was synthesized by mixing **DPEC** and DMAPAPA in 1:4 molar ratio and, thus, conducting the reaction between epoxy groups and hydrogen atoms of the active primary amino groups in DMAPAPA. Mixtures of such a molar ratio do not cross-link, since only individual DMAPAPA molecules bond to each epoxy group, and the formed oligomer remains soluble. In order to form a cross-linked layer additional **DPEC** was added to the original oligomer mixture, resulting in molar ratio of the starting compounds of 3:4, *i.e.* exactly the same as required for cross-linking. Cross-linking in the layer took place in 1 h at 120 °C. Transparent and homogeneous films insoluble in THF were obtained.

The XTOF technique was used to study charge transport properties of the obtained cross-linked films **NET1** and **NET2**. As seen from Figure 6 and Table 1, the hole mobility of **NET1** reaches 10^−4^ cm^2^·V^−1^·s^−1^ at an electric field of 10^6^ V·cm^−1^ and is close to the reported values for the parent 1,3-diphenylethenylcarbazolyl-based compounds [13]. The result is two order of magnitude higher than a well-known hole transporting polymer poly(9-vinylcarbazole) (PVK) (μ_h_ = 10^−6^ cm^2^·V^−1^·s^−1^ at an electric field of 10^5^ V·cm^−1^) [23] and comparable to that of 4,4'-*N*,*N'*-dicarbazolebiphenyl (CBP) (μ_h_ = 10^−3^ cm^2^·V^−1^·s^−1^ at 10^6^ V·cm^−1^) [24]. Although, it has to be noted that mobility results of CBP were obtained from vacuum deposited films, while simpler and cheaper solution processing was used in case of **NET1**. The observed noticeable improvement of the hole mobility in **NET1** over **DPEC** could be attributed to elimination of the polycarbonate from the composition, leading to the increase in concentration of the charge transporting moieties. It is also highly unlikely that crosslinking agent **TBBT**, used in experiments, had any direct influence on the charge mobility results of **NET1**. **TBBT** is not linked directly to the π-conjugated systems of the molecule, furthermore, in previous experiments with similar dimers and polymers we failed to notice a strong correlation between hole mobility and the linking fragment used [25,26,27]. Indirect effects, however, could not be ruled out, as linking fragments certainly affects the way molecules pack in the film. Unfortunately, the attempts to measure hole mobility of **NET2** failed. This may be attributed to the increasing concentration of charge trapping sites with the addition of DMAPAPA and increasing positional and energetic disorder.

## 3. Experimental Section

### 3.1. Materials

All chemicals for synthesis were purchased from Sigma-Aldrich (Taufkirchen, Germany) and TCI Europe (Haven, Belgium) and used as received without further purification. 1,3-Bis(2,2-diphenylethenyl)-9*H*-carbazol-2-ol (**1**), was obtained according to a synthetic procedure described by us previously [13]. DMAPAPA used in cross-linking experiments was purchased from Atofina Chemicals Inc. (Steiheim, Germany). PC-Z was purchased from Mitsubishi Gas Chemical Co. (Tokyo, Japan).

*1,3-Bis(2,2-diphenylethenyl)-2-(oxiran-2-methyl)-9H-carbazole* (**2**). Compound **1** (5.4 g, 0.01 mol) was dissolved in epichlorohydrin (79 mL, 93.0 g, 1 mol). Benzyl triethylammonium chloride (0.23 g, 0.001 mol) was added. The reaction mixture was intensively stirred at reflux temperature for 10 min. After termination of the reaction (TLC, acetone: *n-*hexane, 1:4) the reaction mixture was diluted with chloroform and extracted with distilled water. The organic layer was dried over anhydrous MgSO_4_, filtered and solvents were removed. The obtained colorless oil was purified by column chromatography using acetone: *n-*hexane (3:22, v/v) as the eluent. Obtained crystals were filtered off and washed with 2-propanol. The yield of compound **2** was 4.6 g (77%); m.p.: 214 °C–215 °C. ^1^H-NMR (CDCl_3_, ppm): δ 7.50–6.84 (m, 28H, Ht, Ph, C=CH, NH); 4.28 (dd, *J*_1_ = 11.1 Hz, *J*_2_ = 4.0 Hz, 1H, one proton of OCH_2_CH); 4.16 (dd, *J*_1_ = 11.1 Hz, *J*_2_ = 5.7 Hz, 1H, other proton of OCH_2_CH); 3.39–3.29 (m, 1H, CHO); 2.75 (dd, *J*_1_ = 5.0 Hz, *J*_2_ = 4.1 Hz, 1H, one proton of OCH_2_ from epoxy group); 2.60 (dd, *J*_1_ = 5.0 Hz, *J*_2_ = 2.6 Hz, 1H, other proton of OCH_2_ from epoxy group). IR (KBr, cm^−1^): 3441 (NH); 3049, 3022 (aromatic CH); 2919, 2872 (aliphatic CH); 1596, 1576, 1490, 1475, 1443 (C=C, C-N); 1247, 912, 894, 846 (epoxy); 1208, 1173, 1142, 1075, 1054, 1029 (C-O-C); 809, 768, 749, 733, 697 (CH=CH of 1,2,3,-trisubstituted carbazole, monosubstituted benzene). Anal. Calcd for C_43_H_33_NO_2_: C, 86.17; H, 5.68; N, 2.38. Found: C, 86.69; H, 5.58; N, 2.15.

*4,4ʹ-Bis{3-hydroxy-4-[1,3-bis(2,2-diphenyletenyl)-9H-carbazol-2-oxy]-1-thiabutyl}thio-bisbenzene* (**3**). To the mixture of **2** (2.30 g, 0.004 mol) and 4,4'-thiobisbenzenethiol (0.50 g, 0.002 mol) in THF (20 mL) triethylamine (0.4 mL, 0.001 mol) were added and the obtained mixture was stirred at room temperature overnight. After termination of the reaction (TLC monitoring) THF and trimethylamine were removed and the residue was purified by column chromatography using acetone:*n*-hexane (1:4, v/v) as the eluent. A 20% solution of the resulting product in toluene was prepared and poured with intensive stirring into a 10-fold excess of *n*-hexane. The precipitate was filtered off and washed with *n*-hexane. The yield of compound 3 was 2.37 g (82%). ^1^H-NMR (CDCl_3_, ppm): δ 7.48–6.86 (m, 64H, Ht, Ph, C=CH, NH); 4.21 (d, *J* = 4.7 Hz, 4H, OCH_2_CH); 4.07–3.98 (m, 2H, CHO); 3.12 (dd, *J*_1_ = 13.8 Hz, *J*_2_ = 6.1 Hz, 2H, one proton of SCH_2_); 3.04 (dd, *J*_1_ = 13.8 Hz, *J*_2_ = 6.8 Hz, 2H, other proton of SCH_2_); 2.61 (d, *J* = 4.9 Hz, 2H, OH). IR (KBr, cm^−1^): 3549 (OH); 3423 (NH); 3053, 3021 (aromatic CH); 2921, 2867 (aliphatic CH); 1598, 1576, 1491, 1475, 1443 (C=C, C-N); 1208, 1167, 1098, 1073, 1029, 1010 (C-O-C); 811, 765, 744, 698 (CH=CH of 1,2,3,-trisubstituted carbazole, monosubstituted and 1,4-disubstituted benzene); 641, 611 (C-S). Anal. Calcd for C_98_H_76_N_2_O_4_S_3_: C, 80.09; H, 5.33; N, 1.90. Found: C, 79.83; H, 5.31; N, 1.74.

*4,4ʹ-Bis{3-[1,3-bis(2,2-diphenyletenyl)-9-(oxiran-2-methyl)-9H-carbazol-2-oxy]-3-(oxi-ran-2-methoxy)-1-thiabutyl}thiobisbenzene* (**DPEC**). To a vigorously stirred solution of **3** (2.0 g, 0.001 mol) in epichlorohydrin (6.5 mL, 0.083 mol) anhydrous Na_2_SO_4_ (0.57 g, 0.004 mol) and 85% powdered KOH (1.10 g, 0.017 mol) were added in three equal portions every hour with prior cooling of the reaction mixture to 30 °C. After 4 h at 40 °C the reaction was terminated and the mixture was extracted with ethyl acetate. The organic layer was dried over anhydrous magnesium sulphate and filtered off. Ethyl acetate and excess of epichlorohydrin were removed and the residue was purified by column chromatography using acetone: *n*-hexane (1:4, v/v) as the eluent. A 20% solution of the resulting product in toluene was prepared and poured with intensive stirring into a 10-fold excess of *n*-hexane. The precipitate was filtered off and washed with *n*-hexane. The yield of **DPEC** was 1.46 g (63%). ^1^H-NMR (CDCl_3_, ppm): δ 7.54–6.89 (m, 62H, Ht, Ph, C=CH); 5.23–5.05 (m, 1H, from NCH_2_); 4.76–4.47 (m, 2H, OCH_2_CHS); 4.38–4.23 (m, 1H, from NCH_2_); 4.20–3.85 (m, 4H, OCH_2_CHS); 3.62–3.42 (m, 2H, NCH_2_); 3.37–2.97 (m, 8H, SCH_2_, OCH_2_CHO); 2.83–2.11 (m, 12H, from epoxy). IR (KBr, cm^−1^): 3052, 3021 (aromatic CH); 2996, 2920, 2867 (aliphatic CH); 1594, 1494, 1476, 1443, 1410 (C=C, C-N); 1248, 912 (epoxy); 1183, 1157, 1098, 1066, 1030, 1010 (C-O-C); 812, 766, 745, 698 (CH=CH of 1,2,3,9-tetrasubstituted carbazole, monosubstituted and 1,4-disubstituted benzene); 641, 611 (C-S). Anal. Calcd for C_110_H_92_N_2_O_8_S_3_: C, 79.30; H, 5.57; N, 1.68. Found: C, 79.07; H, 5.41; N, 1.51.

### 3.2. Cross-Linking Experiments

#### 3.2.1. Preparation of **NET1**

The layer samples were coated on ITO from solution of **DPEC** (20 mg) and **TBBT** (6 mg) in 1 mL of THF and heated at 120 °C in a thermostat for 3 h. After cross-linking, solubility of the obtained HTM layer **NET1** was checked by measuring thickness of the layer after treatment with THF. The control sample was stored under room conditions.

#### 3.2.2. Preparation of **NET2**

To DMAPAPA solution in THF (0.212 g, 3.25 mg·mL^−1^) **DPEC** (1.7 mg) was added and the mixture kept at room temperature for 2 h and then at 60 °C for another 2 h. Afterwards, **DPEC** (3.4 mg) in THF (0.140 mL) was added to the obtained intermediate oligomer, and a clear viscous solution was thus obtained. Small layer samples were coated on Al, and heated at 120 °C for 1 h. After cross-linking, solubility of the obtained **NET2** was checked by measuring thickness of the layer after treatment with THF.

### 3.3. Measurements

The ^1^H-NMR spectra were recorded on a Unity Inova (300 MHz) instrument (Varian, Palo Alto, CA, USA) at room temperature. All the data are given as chemical shifts in δ (ppm), (CH_3_)_4_Si (TMS, 0 ppm) was used as an internal standard. IR-spectroscopy was performed on a Spectrum BX II FT-IR System (Perkin Elmer, Walttam, MA, USA) using KBr pellets. The course of the reactions and purity of the products were monitored by TLC on ALUGRAM SIL G/UV254 plates developed with UV light. Silica gel (grade 9385, 230–400 mesh, 60 Å, Aldrich) was used for column chromatography. Elemental analysis was performed with a CE-440 elemental analyzer, Model 440 C/H/N/ (Exeter Analytical, Coventry, UK) Differential scanning calorimetry (DSC) was performed on a Q10 calorimeter (TA Instruments, New Castle, DE, USA) at a scan rate of 10 K·min^−1^ under the nitrogen atmosphere. The glass transition temperatures for the investigated compounds were determined during the second heating scan. A Electrothermal MEL-TEMP capillary melting point apparatus was used for determination of melting points. UV spectra were recorded on a Perkin Elmer Lambda 35 spectrometer. A solution of investigated compound in THF (c = 10^−^^4^ M) and a microcell with an internal width of 1 mm were used. Fluorescence of the investigated compounds was measured by Edinburgh Instruments FS920 spectrometer. A dilute solution of the required compound was prepared by dissolution in spectral-grade toluene (c = 1 × 10^−5^ M).

The ionization potential *I*_p_ of the layers of the synthesized compounds was measured by the electron photoemission in air method, similar to the one used in [28] and described in [19], the measurement error is evaluated as ±0.03 eV. The samples for the ionization potential measurement were prepared by dissolving materials in THF and coating them on Al plates pre-coated with ~0.5 μm thick methylmethacrylate and methacrylic acid copolymer adhesive layer. The thickness of the transporting material layer was 0.5–1 μm.

The hole drift mobility was measured by XTOF technique [29,30]. The samples for hole drift mobility measurements were prepared from 1:1 mass proportion compositions of compounds with polycarbonate (PC-Z) (Iupilon Z-200 from Mitsubishi Gas Chemical Co.). The sample substrate was polyester film with conductive Al layer. Electric field was created by positive corona charging. The charge carriers were generated at the layer surface by illumination with pulses of xenon flash lamp with UV filter (pulse duration was 1 µs, wavelength 330–390 nm). The layer surface potential decrease as a result of pulse illumination was up to 1%–5% of initial potential before illumination. The capacitance probe that was connected to the wide frequency band electrometer measured the speed of the surface potential decrease d*U*/d*t*. The transit time *t*_t_ was determined by the kink on the curve of the d*U*/d*t* transient in double logarithmic scale. The drift mobility was calculated according to the equation μ = *d*^2^/*U*_0_*t*_t_, where d is the layer thickness, *U*_0_—the surface potential at the moment of illumination. The slope coefficients of XTOF transients have been calculated according to the method provided in the literature [31,32].

## 4. Conclusions

A 1,3-diphenylethenylcarbazolyl-based hole transporting material containing four epoxypropyl groups was synthesized and investigated for the development of cross-linked hole transporting layers with high solvent resistance. **DPEC** can be completely cross-linked to form smooth insoluble films through the reaction of the epoxy groups with the aliphatic amine DMAPAPA as cross-linking agent, while **TBBT**, containing two mercapto groups, gave only partly cross-linked ones. The hole drift mobility of the cross-linked films reaches 10^−4^ cm^2^·V^−1^·s^−1^ at an electric field of 10^6^ V·cm^−1^.
